# Pulsed radiofrequency on peripheral nerve as a rehabilitation aid

**DOI:** 10.1186/s44158-024-00156-4

**Published:** 2024-03-19

**Authors:** Giulia Bongiorno, Helena Biancuzzi, Francesca Dal Mas, Rym Bednarova, Alessandro Vittori, Luca Miceli

**Affiliations:** 1 Friuli Riabilitazione Rehabilitation Center, City of Roveredo in Piano, Pordenone, Italy; 2 Department of Economics, Ca’ Foscari University of Venice, Venice, Italy; 3 Department of Management, Ca’ Foscari University of Venice, Venice, Italy; 4 Collegium Medicum, University of Social Sciences, Lodz, Poland; 5 Pain Medicine, Hospital of Latisana, Udine, Latisana Italy; 6 Department of Anesthesia and Critical Care, ARCO Roma, Ospedale Pediatrico Bambino Gesù IRCCS, Rome, Italy; 7 Department of Pain Medicine, IRCCS C.R.O. National Cancer Institute of Aviano, Pordenone, Aviano Italy

**Keywords:** Chronic pain, Pulsed radiofrequency, Rehabilitation, Fatigue

## Abstract

The work described below explores the field of the effects of pulsed radiofrequency for pain relief purposes. While the effects of this technique on pain modulation (A-delta and C fibers) are relatively well-known, little has been written yet about the potential of pulsed radiofrequency interactions with other fibers. The proposed algorithm, specifically elaborated, investigates the effect of this technique on neuromuscular fatigue, through a surface electromyographic study of the femoral nerve of a patient with residual pain after knee arthroplasty surgery, before and after the treatment. This work yields a preliminary result that is encouraging for subsequent large-scale studies.

Pulsed radiofrequency is a neuromodulation technique applied clinically since the 1990s. It is still a technique widely used in various areas of pain medicine in the musculoskeletal field thanks to its effectiveness and safety [1]. However, there is currently no exact knowledge of how this method works. However, there are several theories, mainly in the influence area on ion channels, modification of neurotransmitters, actions on microglia, inflammatory cytokines, and other additional mechanisms [2]. The authors reflected on different aspects, considering pain therapy and rehabilitation as activities that work in synergy to restore the damaged function of the patients. First, the working group wondered whether pulsed radiofrequency could have other effects on the peripheral nerve, in addition to the effects on the modulation of the conduction of the painful stimulus to the brain. Secondly, the authors wondered whether the nerve treated with pulsed radiofrequency maintains its neuromuscular recruitment capabilities unchanged. This data is very relevant: if there was a reduction in this capacity, the rehabilitation process, especially after a trauma or injury, could be slowed down. In this case, the advantage of good pain control could disappear. A simple and promising analysis tool to study fatigue resistance is present in the literature. The topic is treated in the field of muscle fatigue in athletes and uses surface electromyography [3]. Surface electromyography is a non-invasive method capable of evaluating the electrical activity of muscle fibers over which wireless probes are placed. The study of muscle fatigue is one of its uses. This consists of measuring the amount of electrical energy of a given neuro-muscular complex necessary to maintain an isometric contraction for a certain period (normally 30–60 s). This value is generally indexed and expressed as a percentage, relating it to the electrical energy measured during a maximum voluntary isometric contraction (MVIC) maintained for 3–5 s. Thus, to compare data obtained from different subjects or from the same subjects at different times [4]. The smaller the amount of electrical energy used for this exercise (RMS—root mean square compared to MVIC) and the greater the resistance to fatigue of the neuromuscular complex investigated an indicator of good functioning of the system [3]. The previously mentioned algorithm was tested on a 79-year-old patient. The measurements were taken before the pulsed radiofrequency treatment, and 4 weeks after the treatment. The patient underwent total knee arthroplasty, for severe gonarthrosis, 12 months before treatment. The latter was performed at the pain medicine clinic of the Cro Institute of Aviano (Italy). The treatment was performed at level of adductor canal; we chose this approach to better appreciate any reductions in the functionality of the quadriceps muscle (motor fibers), which we could not have well appreciated by working at the level of the geniculate nerves. No side effects were recorded. We used a 50-mm needle, sterility, and with continuous ultrasound guidance. Duration of pulsed radiofrequency (performed with TherMedico NK 1 radiofrequency lesion generator, Hesse, Germany) was 5 min at 35 V, with a needle tip temperature of 42 °C, according to experiences already published in the literature [4]. The effectiveness of the treatment, measured three weeks after the procedure, was documented by a reduction in the NRS (Numerical Rate Scale) at movement/rest from 8/5 to 3/0. For the kinematics analysis, we used bipolar pre-gelled electrodes (Ag/AgCl) with a diameter of 10 mm and the distance between the electrodes centers was 2 cm. The surface electrodes were placed on the belly of the right rectus femuris muscle, along the midline longitudinal axis of the muscle. The raw EMG, acquired from Freemg 1000 BTS probes (BTS Bioengineering, Garbagnate Milanese, Italy), were processed into a value root mean square (RMS) with a 50 ms window. A 20–450 Hz band filter was used. Signal processing and EMG analysis were performed using Emg-analyze software (BTS Bioengineering, Garbagnate Milanese, Italy). The change in energy cost in isometric maintenance (% RMS/MVIC) detected on the right rectus femoris muscle (in Fig. 1 an example of electrode positioning on a healthy subject) went from 80 to 59.8. This variation therefore showed an improvement in the patient’s performance, without there having been a physiotherapeutic approach between the two measurements. The latter would have been impossible as the patient could not go to a rehabilitation studio. The improvement observed may be due to better resistance to fatigue related to less contracture of the quadriceps muscle observed in the patient after treatment. This phenomenon can be due to the pain relief but also it could be a direct effect of the pulsed radiofrequency treatment. This also improved the antalgic lameness, potentially making the future work of the physiotherapist more profitable [5, 6]. This first experience, limited to a single subject, although without being able to make definitive statements in an absolute sense as it is a single observation, offers the scientific community an innovative non-invasive analysis tool for subsequent studies aimed at confirming the harmlessness of pulsed radiofrequency on the capacity of neuromuscular recruitment of patients and to better investigate its mechanisms of action which are still partly unknown. Another possible limitation of this work may be due to the fact that the proposed kinematic analysis protocol, although non-invasive and not expensive, requires about five minutes to be performed and a specific know how and technical equipment.

**Fig. 1:**
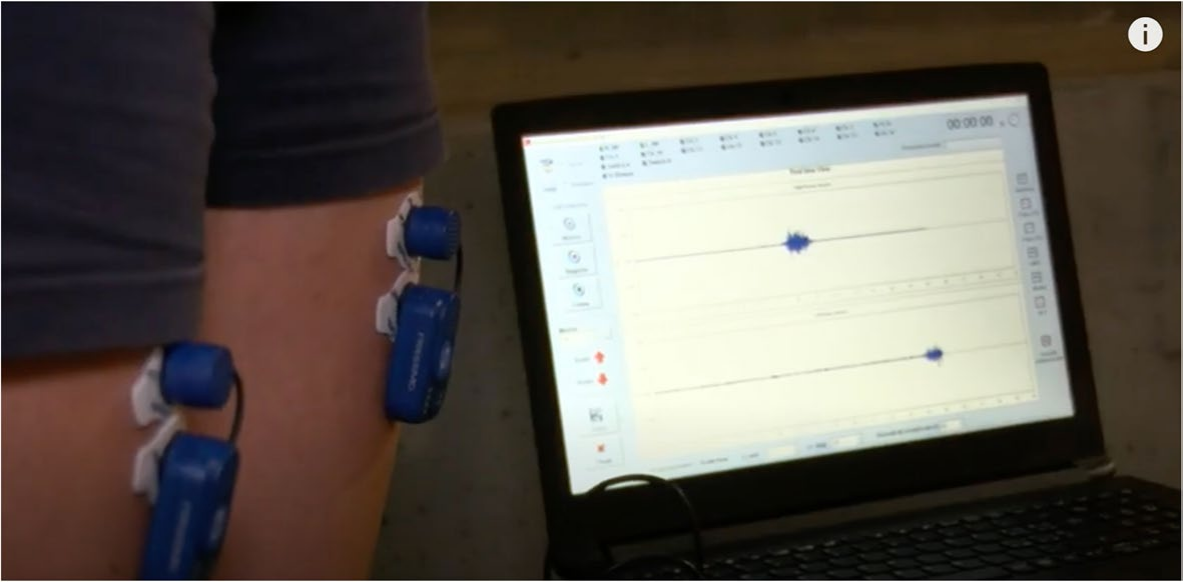
Example of placement of surface electromyographic probes on the rectus femoris muscles, connected to a PC in wireless mode

## Data Availability

No datasets were generated or analysed during the current study.
